# Study on brain damage patterns of COVID-19 patients based on EEG signals

**DOI:** 10.3389/fnhum.2023.1280362

**Published:** 2023-11-24

**Authors:** Yang Yao, Yingnan Liu, Yu Chang, Zihan Geng, Xingting Liu, Songnan Ma, Zhiyun Wang, Chenguang Zheng, Jiajia Yang, Dong Ming

**Affiliations:** ^1^Department of Neurology, Tianjin First Central Hospital, Tianjin, China; ^2^Institute of Medical Engineering and Translational Medicine, Tianjin University, Tianjin, China; ^3^Tianjin Key Laboratory of Brain Science and Neural Engineering, Tianjin University, Tianjin, China

**Keywords:** COVID-19, electroencephalography, functional connectivity network, sample entropy, directed transfer function

## Abstract

**Objective:**

The coronavirus disease 2019 (COVID-19) is an acute respiratory infectious disease caused by the SARA-CoV-2, characterized by high infectivity and incidence. Clinical data indicates that COVID-19 significantly damages patients’ perception, motor function, and cognitive function. However, the electrophysiological mechanism by which the disease affects the patient’s nervous system is not yet clear. Our aim is to investigate the abnormal levels of brain activity and changes in brain functional connectivity network in patients with COVID-19.

**Methods:**

We compared and analyzed electroencephalography signal sample entropy, energy spectrum, and brain network characteristic parameters in the delta (1–4 Hz), theta (4–8 Hz), alpha (8–13 Hz), and beta (13–30 Hz) bands of 15 patients with COVID-19 and 15 healthy controls at rest.

**Results:**

At rest, energy values of the four frequency bands in the frontal and temporal lobes of COVID-19 patients were significantly reduced. At the same time, the sample entropy value of the delta band in COVID-19 patients was significantly increased, while the value of the beta band was significantly decreased. However, the average value of the directed transfer function of patients did not show any abnormalities under the four frequency bands. Furthermore, node degree in the temporal lobe of patients was significantly increased, while the input degree of the frontal and temporal lobes was significantly decreased, and the output degree of the frontal and occipital lobes was significantly increased.

**Conclusion:**

The level of brain activity in COVID-19 patients at rest is reduced, and the brain functional network undergoes a rearrangement. These results preliminarily demonstrate that COVID-19 patients exhibit certain brain abnormalities during rest, it is feasible to explore the neurophysiological mechanism of COVID-19’s impact on the nervous system by using EEG signals, which can provide a certain technical basis for the subsequent diagnosis and evaluation of COVID-19 using artificial intelligence and the prevention of brain nervous system diseases after COVID-19 infection.

## 1 Introduction

The coronavirus disease 2019 (COVID-19) is an infectious disease caused by severe acute respiratory syndrome coronavirus 2 (SARS-CoV-2). Since its first outbreak in Wuhan, Hubei Province, China at the end of 2019, it has rapidly spread worldwide. This virus has triggered a global public health crisis and has had a huge impact on the global healthcare system and socio-economy. As the number of COVID-19 cases increases, clinical research has gradually found that the virus not only affects the respiratory system but may also have adverse effects on the central nervous system (Varatharaj et al., 2020). With the increasing reports of neurological manifestations of SARS-CoV-2 infection, researchers use brain electroencephalography (EEG) to detect patients (Petrescu et al., 2020). However, the number of existing articles is still small and lacks control groups; therefore, it is necessary to investigate such neurological abnormalities using EEG in patients with COVID-19.

In recent years, several studies have explored the changes of EEG characteristics in patients with COVID-19. Research reported that COVID-19 infection may cause changes in EEG patterns and wave amplitudes, suggesting that COVID-19 may have an effect on brain activity (Pasini et al., 2020). Pastor et al. (2020) observed that temporal lobes showed different distribution for EEG bands in COVID-19 patients. Additionally, Shannon’s spectral entropy was higher, and hemispheric connectivity was lower for COVID-19 patients (Pastor et al., 2020). The possible causes of EEG abnormalities include inflammatory damage, hypoxemia, or direct damage to brain neurons caused by the virus. It was found that EEG signal amplitudes significantly increased in patients with epilepsy and moderate pneumonia, indicating that COVID-19 may affect EEG signals. The study found that in patients with hypoxemia, EEG theta frequency band enhancement and alpha, beta frequency band attenuation correlation (Wu et al., 2020). Another study conducted on an individual who recovered from COVID-19 showed that the characteristics of EEG signals changed over time, indicating that viral infection may have long-term effects on the central nervous system (Zanin et al., 2022). However, the current research only focused on the changes of brain wave shape and did not conduct in-depth exploration, so the electrophysiological mechanism of nervous system injury in patients with COVID-19 is still unclear.

To investigate changes in brain activity and abnormal phenomena in the brains of COVID-19 patients, this study preprocessed the resting-state EEG signals of COVID-19 patients and healthy control group. Sample entropy was used to calculate the complexity of the EEG signals, indirectly reflecting the activity levels of the two groups. Energy spectra were used to reflect the activity states of various brain regions. The directed transfer function (DTF) matrix was selected to reflect the causal connection strength between the cortical regions. A brain network model was constructed using the DTF matrix, and graph theory was used for quantitative analysis of the brain network to explore the mechanism of virus impact on brain electrical activity and understand the indirect effects of the COVID-19 on the central nervous system.

## 2 Materials and methods

### 2.1 Participants

15 patients with COVID-19 patients took part in the study, with 15 healthy subjects as controls. Demographic and clinical features of patients are reported in Table 1. All participants in this study underwent EEG collection at the Neurology Department of the First Central Hospital in Tianjin. EEG signals of patients with COVID-19 were collected 28 days after COVID-19 infection, when the patients were at the stage of mild or moderate disease, the distinction between mild and moderate patients were made by the Tianjin COVID-19 Treatment Expert Group according to the symptoms and CT manifestations of the patients, all patients were vaccinated with COVID-19 vaccine, and were given regular symptomatic and traditional Chinese medicine treatment within 14 days after COVID-19 infection. The healthy control group had no history of serious neurological diseases, mental illnesses, or use of psychotropic drugs. Before collecting EEG data, the healthy group carried out the nucleic acid testing, the results showed that they were not infected with COVID-19. And the healthy group had no history of COVID-19 infection. Informed consent was obtained from all participants. This study follows the Declaration of Helsinki and has been approved by the Ethics Committee of Tianjin First Central Hospital. All participants have signed informed consent forms.

**TABLE 1:** Demographic information by clinical status.

Demographics	COVID-19	Healthy	Test statistic (df)	*P*-value
*N*	15	15	–	–
Age(y)	47.80(3.56)	29.00(3.70)	*U* = 67.50	0.002
Sex (%Male)	53.33% (8)	33.33% (5)	X^2^(1) = 1.22	0.269
MMSE	25.20 (0.82)	–	–	–
AIS	4.85 (0.70)	–	–	–
**Comorbidities**				
Hypertension	26.67% (4)	–	*U* = 82.50	0.217
Hyperlipidemia	20.00% (3)	–	*U* = 90.00	0.367
Diabetes	13.33% (2)	–	*U* = 97.50	0.539
Genetic history	6.67% (1)	–	*U* = 105.00	0.775
Coronary heart disease	6.67% (1)	–	*U* = 105.00	0.775
Chronic respiratory disease	6.67% (1)	–	*U* = 105.00	0.775
Chronic kidney disease	6.67% (1)	–	*U* = 105.00	0.775

Values are mean ± SEM or % (N). Mini Mental Status Examination (MMSE) ranges from 0 (worst) to 30 (best). Athens Insomnia Scale (AIS) with a total score of <4 indicates no sleep disorders, 4–6 indicates suspected insomnia, and a total score of >6 indicates insomnia. MMSE, Mini Mental Status Examination; AIS, Athens Insomnia Scale.

### 2.2 EEG recording and preprocessing

During the resting state, EEG data were collected from 30 participants using 8 electrodes (Fp1, Fp2, T3, T4, C3, C4, O1, O2) to record activity in the frontal, temporal, central, and occipital regions. The data were collected in a quiet and comfortable experimental environment, ensuring stable connections between the EEG amplifier and electrodes. The participants’ scalps were in close contact with the electrodes and ground wire through conductive media such as electrode gel or saline solution to ensure the quality of the EEG signals. The collection instrument used a BE Micro dynamic electroencephalogram recorder and a NCC amplifier, the electrodes were positioned according to the international 10/20 system, with a sampling frequency of 125Hz and impedance maintained below 10kΩ. Each resting state experiment lasted for 5 min with participants’ eyes closed.

Preprocessing the recorded EEG data used the EEGLAB toolbox (V2021.1) based on the MATLAB platform.

(1) 1∼30 Hz bandpass filtering, mainly removing high-frequency interference components and divided the data into four frequency bands: delta (1–4 Hz), theta (4–8 Hz), alpha (8–13 Hz), and beta (13–30 Hz).
(2) Using independent component analysis (ICA) to remove interference signals such as electromyography and electrocardiogram.
(3) Using rejection bad channel and epoch method to replace some channels with imperfect signal recording.

### 2.3 Calculation of EEG features

#### 2.3.1 Energy

The energy of a signal in the (−∞, +∞) interval of time:


(1) $$E=\lim_{T\to \infty }\int_{-T}^{T}{\left|f\,\left(t\right)\right|}^{2}\,dt$$

#### 2.3.2 Sample entropy (SampEn)

SampEn measures the complexity of a time series by the probability of new patterns being generated in the signal (Liu et al., 2016).

For a time series composed of N data, *X* = *x*_1_, *x*_2_, …, *x*_*N*_. The calculation method for SampEn is as follows:

(1) Form a set of vectors $X_{m}^{1},\ldots ,X_{m}^{N-m+1}$, for 1 ≤ *i* ≤ *N* − *m* + 1, it is defined as:


(2) $$X_{m}^{i}=\left(x_{i},x_{i+1},x_{i+m-1}\right)$$

(2) Define the distance between vectors $X_{m}^{i}$ and $X_{m}^{j}$ as the maximum absolute difference between their respective scalar components:


(3) $$d\,\left[X_{m}^{i},X_{m}^{j}\right]=\max_{k=0,\ldots ,m-1}\left|x_{i+k}-x_{j+k}\right|$$

(3) For a given $X_{m}^{i}$, count the number of *j*(1 ≤ *j* ≤ *N* − *m*, *j* ≠ *i*), denote as *B_i_*, such that $d\,\left[X_{m}^{i},X_{m}^{j}\right]\leq r$, that is, *B_i_* is the number of $d\,\left[X_{m}^{i},X_{m}^{j}\right]\leq r,j\neq i$. Then, for 1 ≤ *j* ≤ *N* − *m*,


(4) $$B_{i}^{m}\,\left(r\right)=\frac{1}{N-m-1}\times B_{i}$$

(4) Define *B^m^*(*r*) as


(5) $$B^{m}\,\left(r\right)=\frac{1}{N-m}\,\sum_{i=1}^{N-m}B_{i}^{m}\,\left(r\right)$$

(5) Similarly, calculate $A_{i}^{m}\,\left(r\right)$ as 1/(*N* – *m* + 1) times the number of *j*(1 ≤ *j* ≤ *N* − *m*, *j* ≠ *i*), such that the distance between $X_{m+1}^{j}$ and $X_{m+1}^{i}$ is less than or equal to:


(6) $$A_{i}^{m}\,\left(r\right)=\frac{1}{N-m-1}\times A_{i}$$

Set *A^m^*(*r*) as


(7) $$A^{m}\,\left(r\right)=\frac{1}{N-m}\,\sum_{i=1}^{N-m}A_{i}^{m}\,\left(r\right)$$

Thus, *B^m^*(*r*) is the probability that two sequences will match for *m* points, whereas *A^m^*(*r*) is the probability that two sequences will match for *m*+1 points.

(6) Finally, define


(8) $$S\,a\,m\,p\,E\,n\,\left(m,r\right)=\lim_{N\to \infty }\left\{-\ln\left[\frac{A^{m}\,\left(r\right)}{B^{m}\,\left(r\right)}\right]\right\}$$

Which is estimated by the statistic


(9) $$S\,a\,m\,p\,E\,n\,\left(m,r,N\right)=-\ln\left[\frac{A^{m}\,\left(r\right)}{B^{m}\,\left(r\right)}\right]$$

In this study, let *m* = 2 and *r* = 0.2.

#### 2.3.3 Directed transfer function (DTF)

In this study, the connectivity measures monitored the functional connectivity of the EEG signals. DTF is a measure based on Granger Causality, but defined in the frequency domain.

(1) Assuming that the original EEG signal is a matrix of M-channels:


(10) $$Y\,\left(n\right)={\left[y_{1}\,\left(n\right),\ldots ,y_{M}\,\left(n\right)\right]}^{T}$$

In the equation, each vector represents the sequence of EEG data corresponding to the lead.

(2) Establishing a P-order multivariate autoregressive model (MYAR) based on Y (t), whose formula is:

The order P in the equation is determined based on the Bayesian information criterion, where A_*r*_ is the coefficient matrix of size M*M, and E(n) is the error between the current value and the predicted value.


(11) $$Y\,\left(n\right)=\sum_{r=1}^{p}A_{r}\,Y\,\left(n-r\right)+E\,\left(n\right)$$

(3) Perform fourier transform on the coefficient matrix, i.e.,:


(12) $$A\,\left(f\right)=I-\sum_{r=1}^{p}A_{r}\,e^{-i\,2\,\pi \,f\,r}$$

where I is an M-dimensional identity matrix.

(4) The DTF value from lead j to lead i is defined as:


(13) $$D\,T\,F_{j\to i}\,\left(f\right)=\frac{\left|H_{i\,j}\right|}{\sqrt{\sum_{k}{\left|H_{k\,j}\right|}^{2}}}$$

*DTF*_*j*→*i*_ represents the ratio of information flowing from lead j to i to all information flowing into i. The DTF value is a normalized value, ranging from [0,1]. The larger the value, the stronger the causal relationship between the two leads.

This research took 8 electrodes as nodes, using the information flow strength (the DTF matrix) as the edge, and the direction of information flow as the direction of the edge.

#### 2.3.4 Constructing binary brain networks

Not all of the weighted links in the original connectivity matrices are significant, and it is necessary to remove the non-significant ones and minimize the noise level. Network binarization can be a good solution to this problem; however, there is no unique strategy for binarizing the connectivity matrices. In this study, we utilized the uniform threshold method to construct the binary network of the cerebral cortex (Jalili, 2016). We applied a threshold T, such that if a link had a weight higher than T, the corresponding entry of the adjacency matrix was set to one, and zero otherwise. During the binarization process, it is important to ensure that the density of the brain network is between 0.3 and 0.8, and that there are no isolated nodes in the network. To achieve the above requirements, we have selected a threshold of 0.0200 for the delta band, 0.0240 for the theta band, 0.0214 for the alpha band, and 0.0146 for the beta band 0.2.3.5 Graph theory metrics.

Through graph theory, any level of network can be abstracted as a set of nodes and edges. In graph theory, the connection between nodes in the network is described by the adjacency matrix.

(1) Node Degree (DEG)


(14) $$D\,\left(i\right)=\sum_{j\,\epsilon \,V}a_{i\,j}+\sum_{j\,\epsilon \,V}a_{j\,i}$$

The total number of connections between a node in the network and other nodes is defined as node degree:

Among them, N is the number of nodes, V is the set of nodes, and *a*_*ij*_ represents the connection from node i to node j in a binary matrix, *a*_*ij*_ represents the connection from node j to node i. Node degree characterizes the importance of nodes in a network.

(2) Input Degree (ID)


(15) $$i\,D\,\left(i\right)=\sum_{j\,\epsilon \,V}a_{i\,j}$$

The number of connections from other nodes to a certain node:

The larger the iD (i), the higher the impact of other nodes on this node.

(3) Output Degree (OD)


(16) $$o\,D\,\left(i\right)=\sum_{j\,\epsilon \,V}a_{j\,i}$$

The number of connections from a node to other nodes:

The larger the oD (i), the higher the impact of this node on other nodes.

### 2.4 Statistic analysis

Statistical tests were performed using the Statistics-and-Machine-Learning Toolbox in MATLAB (version 2022b, MathWorks, Inc. Natick, MA), IBM SPSS Statistics 26.0.0.0 (version 2019, IBM, MA). All figures are expressed as mean ± SEM. After the normality test, the data was all normally distributed, the two-way repeated ANOVA was applied to comparisons between two groups. After multiple heavy tests, a *P*-value < 0.05 was deemed statistically significant.

## 3 Results

### 3.1 The level of brain activity reduced in patients with COVID-19

#### 3.1.1 The energy of prefrontal cortex and temporal cortex in patients with COVID-19 is decreased

**FIGURE 1:**
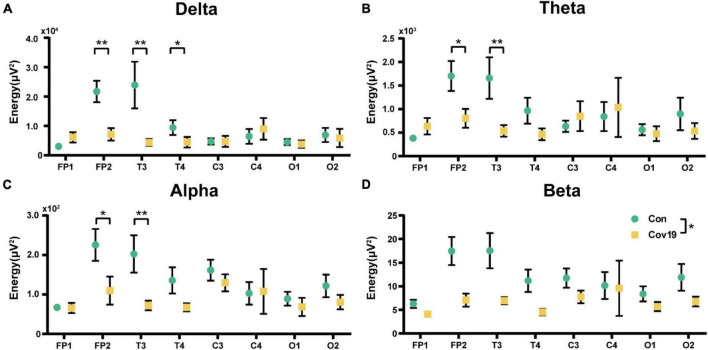
The energy of each lead in the four frequency bands in the resting state: comparison between cov19 patients (cov19) and healthy controls (Con). The horizontal axis has 18 leads, and the vertical axis represents the resting state energy (mean ± SEM). **(A)** Delta band. **(B)** Theta band. **(C)** Alpha band. **(D)** Beta band. Asterisks indicate significant differences between the different intervals; *P < 0.05, **P < 0.01.

We first calculated the energy distribution of various brain regions in resting state for two groups of subjects. The results showed that in four frequency bands, the energy values in FP2, T3, and T4 leads of COVID-19 patients were significantly reduced (Figure 1, two-way repeated ANOVA; delta, main effect of group: *P* = 0.088; main effect of lead: *P* = 1.042 × 10 ^–5^; group × lead interaction: *P* = 9.100 × 10^–6^; theta, main effect of group: *P* = 0.255; main effect of lead: *P* = 0.016; group × lead interaction: *P* = 0.017; alpha, main effect of group: *P* = 0.081; main effect of lead: *P* = 1.741 × 10^–4^; group × lead interaction: *P* = 0.027; beta, main effect of group: *P* = 0.035; main effect of lead: *P* = 0.001; group × lead interaction: *P* = 0.054).

#### 3.1.2 The self-similarity of EEG signals in patients with COVID-19 is abnormal

**FIGURE 2:**
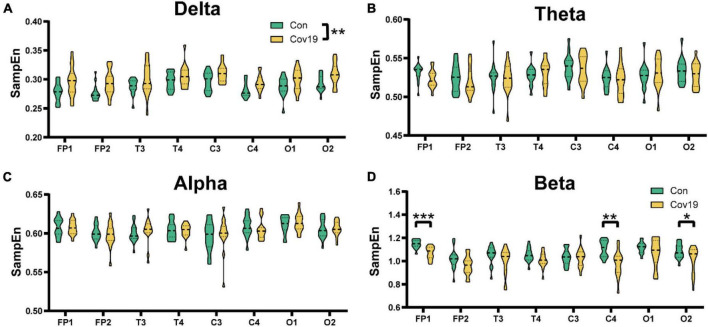
The sample entropy (SampEn) of each lead in the four frequency bands in the resting state: comparison between cov19 patients (cov19) and healthy controls (Con). The horizontal axis has 18 leads, and the vertical axis represents the resting state SampEn (mean ± SEM). **(A)** Delta band. **(B)** Theta band. **(C)** Alpha band. **(D)** Beta band. Asterisks indicate significant differences between the different intervals; *P < 0.05, **P < 0.01, ***P < 0.001.

We further calculated the SampEn of various brain regions in resting state for two groups of subjects. The results showed that the patient group had a significant increase in delta frequency band compared to the healthy control group, indicating a widespread increase in delta frequency band (Figure 2A, two-way repeated ANOVA; delta, main effect of group: *P* = 0.006; main effect of lead: *P* = 2.029 × 10 ^–8^; group × lead interaction: *P* = 0.912). However, there were no significant changes in theta and alpha frequency bands, while the SampEn of patient’s EEG signal in the beta frequency band decreased compared to the control group, with significant differences in Fp1, C4, and O2 (Figures 2B–D, two-way repeated ANOVA; theta, main effect of group: *P* = 0.522; main effect of lead: *P* = 0.001; group × lead interaction: *P* = 0.803; alpha, main effect of group: *P* = 0.929; main effect of lead: *P* = 1.053 × 10^–6^; group × lead interaction: *P* = 0.867; beta, main effect of group: *P* = 0.006; main effect of lead: *P* = 1.322 × 10^–10^; group × lead interaction: *P* = 0.048).

### 3.2 The brain networks of COVID-19 patients undergo reorganization

#### 3.2.1 The functional connectivity between different brain regions in COVID-19 patients is normal

**FIGURE 3:**
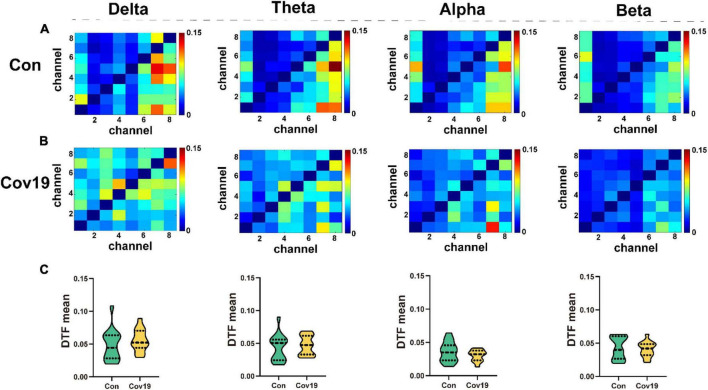
The values of DTF of each lead in the four frequency bands of the two groups of subjects in the resting state. **(A)** DTF matrix heat map of four frequency bands in the healthy control group (Con). **(B)** DTF matrix heat map of four frequency bands in the patient group (Cov19). **(C)** Comparison of mean DTF values in different frequency bands between patients with COVID-19 and healthy control group. Asterisks indicate significant differences between the different intervals.

We calculated DTF connectivity matrices for two groups of participants across four frequency bands. Each row or column of the DTF matrix corresponds to a different node, with each element representing an edge. For this study, we selected eight leads as nodes, resulting in a matrix size of 8x8. We computed the average DTF matrix heatmaps for 15 healthy participants (Figure 3A) and 15 patient participants (Figure 3B), as well as the mean values of all elements in the DTF matrices for both groups and compared them. The results (Figure 3C, student’s *t*-test; delta, *P =* 0.329; theta, *P =* 0.614; alpha, *P =* 0.683; beta, *P =* 0.200) showed no significant differences in functional connectivity strength between brain regions in patients during the resting state.

#### 3.2.2 The core nodes of the brain network in COVID-19 patients have been found to shift

**FIGURE 4:**
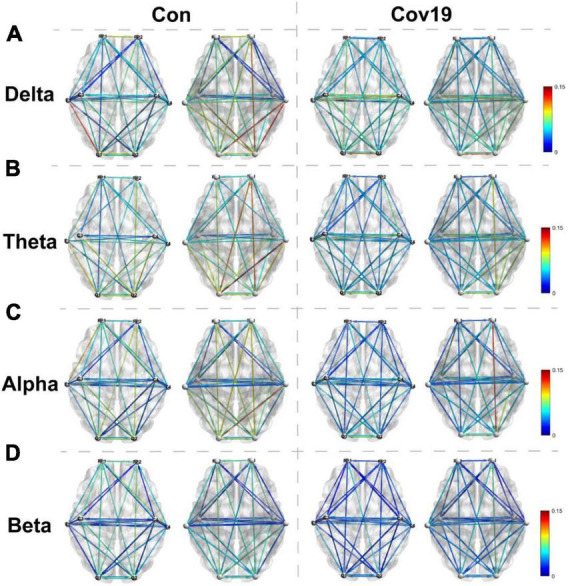
Visual thermogram of functional connectivity different regions of cerebral cortex at different frequency bands in patients with COVID-19 (Cov19) and healthy control group (Con) under resting state. To eliminate false connections, the connection weights between channels greater than the optimal T were retained. Each column displays the top and bottom views of the brain network connection diagram for both the healthy control group and the COVID-19 patient group. **(A)** Delta band, best *T* = 0.0200. **(B)** Theta band, best *T* = 0.0240. **(C)** Alpha band, best *T* = 0.0214. **(D)** Beta band, best *T* = 0.0146.

Two groups of resting-state brain network models for each frequency band were established based on the DTF connection matrix obtained in section “3.2.1. The functional connectivity between different brain regions in COVID-19 patients is normal” (Figure 4). After eliminating false connections using a threshold, local parameters (node degree, in-degree, and out-degree) were calculated using graph theory at the optimal threshold for resting state.

**FIGURE 5:**
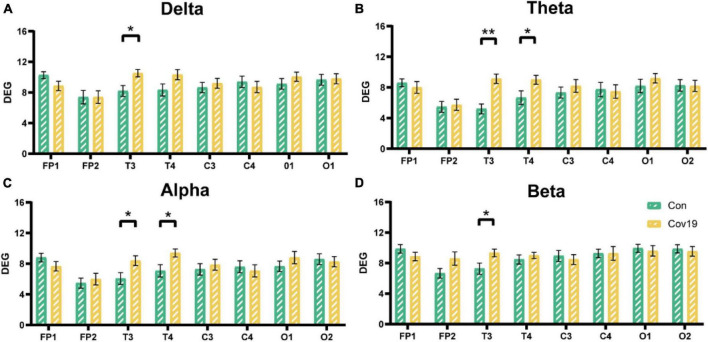
The node degree of each lead in the four frequency bands in the resting state: comparison between cov19 patients (cov19) and healthy controls (Con) with best T. The horizontal axis has 18 leads, and the vertical axis represents the values of degree (DEG, mean ± SEM). **(A)** Delta band, best *T* = 0.0200. **(B)** Theta band, best *T* = 0.0240. **(C)** Alpha band, best *T* = 0.0214. **(D)** Beta band, best *T* = 0.0146. Asterisks indicate significant differences between the different intervals; *P < 0.05, **P < 0.01.

**FIGURE 6:**
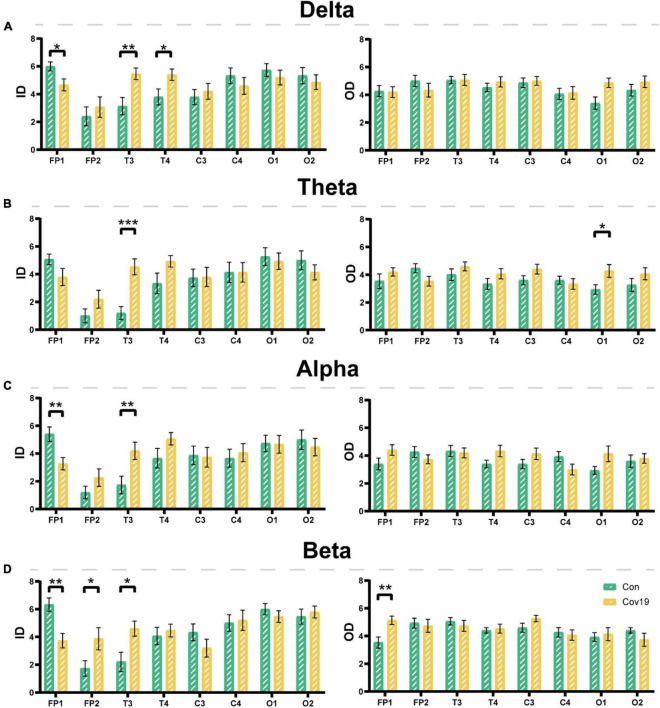
The input degree (ID) and output degree (OD) of each lead in the four frequency bands in the resting state: comparison between cov19 patients (cov19) and healthy controls (Con) with best T. The horizontal axis has 18 leads, and the vertical axis represents the values of in and out degrees (mean ± SEM). **(A)** Delta band. The left side represents the input degree, and the right side represents the output degree. Best *T* = 0.0200. **(B)** Theta band. The left side represents the input degree, and the right side represents the output degree. Best *T* = 0.0240. **(C)** Alpha band. The left side represents the input degree, and the right side represents the output degree. Best *T* = 0.0214. **(D)** Beta band. The left side represents the input degree, and the right side represents the output degree. Best *T* = 0.0146. Asterisks indicate significant differences between the different intervals; *P < 0.05, **P < 0.01, ***P < 0.001.

Node degree can identify the core nodes of the brain functional network. We first calculated the node degree of the two groups of subjects and found that the node degree of the T3 and T4 leads in patients increased significantly in the four frequency bands (Figure 5, two-way repeated ANOVA; delta, main effect of group: *P* = 0.512; main effect of lead: *P* = 6.677 × 10^–5^; group × lead interaction: *P* = 0.002; theta, main effect of group: *P* = 0.236; main effect of lead: *P* = 2.194 × 10^–6^; group × lead interaction: *P* = 0.001; alpha, main effect of group: *P* = 0.366; main effect of lead: *P* = 2.078 × 10^–5^; group × lead interaction: *P* = 0.012; beta, main effect of group: *P* = 0.608; main effect of lead: *P* = 0.001; group × lead interaction: *P* = 0.027), indicating that the mutual connections between the temporal lobe and other brain regions in patients were enhanced. To investigate the reason underlying this enhancement, we further calculated the in-degree and out-degree of each node (Figure 6, two-way repeated ANOVA; ID: delta, main effect of group: *P* = 0.512; main effect of lead: *P* = 2.700 × 10^–6^; group × lead interaction: *P* = 0.006; theta, main effect of group: *P* = 0.236; main effect of lead: *P* = 8.346 × 10^–8^; group × lead interaction: *P* = 0.002; alpha, main effect of group: *P* = 0.366; main effect of lead: *P* = 5.468 × 10^–6^; group × lead interaction: *P* = 0.013; beta, main effect of group: *P* = 0.608; main effect of lead: *P* = 1.858 × 10^–7^; group × lead interaction: *P* = 3.678 × 10^–4^; OD: delta, main effect of group: *P* = 0.512; main effect of lead: *P* = 0.009; group × lead interaction: *P* = 0.061; theta, main effect of group: *P* = 0.236; main effect of lead: *P* = 0.100; group × lead interaction: *P* = 0.004; alpha, main effect of group: *P* = 0.366; main effect of lead: *P* = 0.268; group × lead interaction: *P* = 0.006; beta, main effect of group: *P* = 0.608; main effect of lead: *P* = 0.004; group × lead interaction: *P* = 0.010) and found that the information flow into the FP1 lead in patients decreased significantly compared to the healthy control group, while the information flow into the T3 and T4 leads increased significantly. The results of out-degree showed that the information flow out of the O1 and FP1 leads in patients increased significantly.

## 4 Discussion

Since the outbreak of the novel coronavirus epidemic in late 2019, there has been widespread concern about its severe damage to the respiratory system. The SARS-CoV-2 virus invades the human respiratory system and enters the body through the ACE2 receptor, which exists in various organs, including the brain (Zhao et al., 2020). Subsequent clinical studies have found that COVID-19 not only manifests as respiratory symptoms but also causes damage to multiple organs and systems, such as the heart (Sidik, 2022), liver (Fernandes et al., 2023), kidneys (Gao et al., 2021), eyes (Jeong et al., 2022), and brain (Douaud et al., 2022). In the field of neuroscience, Mao et al. (2020) study had shown that approximately 36.4% of COVID-19 patients experience neurological symptoms, such as headaches, dizziness, consciousness disorders, acute cerebrovascular disease, ataxia, epilepsy, and neuromuscular damage (Mao et al., 2020). Furthermore, experimental evidence suggests that impaired mental and cognitive function in patients is related to changes in brain neurophysiological data (Fernández-Castañeda et al., 2022).

Galanopoulou et al. (2020) observed changes in dominant frontal brain sharp waves, but did not identify the cause of these abnormalities. A recent study suggested that patients infected with COVID-19 exhibited reduced thickness and tissue contrast of gray matter in the orbitofrontal cortex and par hippocampal gyrus. Researchers believe that this phenomenon is related to a reduction in brain cells in areas that control emotion and memory (Douaud et al., 2022). Our study found that the energy of the frontal and temporal lobes in patients with COVID-19 was significantly reduced in four frequency bands, which is consistent with the findings of De Stefano et al. (2020). The study identified focal monomorphic theta slowing in the bilateral frontal-central regions and suggested that EEG can detect neurological dysfunctions in the ICU, even in situations where respiratory ailments are severe (De Stefano et al., 2020).

In this work, we found a significant increase in the complexity of the delta frequency band. This may be related to the generalized rhythmic delta activity observed in Chen’s study through EEG recordings (Chen et al., 2020). Furthermore, a study reported that 16 out of 18 (88.9%) patients showed generalized EEG slowing, with a prevalence of slow waves noted in the anterior (bifrontal) region in 10 out of 18 (55.6%) cases (Cecchetti et al., 2020). These phenomena indicate an increase in the complexity of the patient’s brainwave activity in delta frequency band, reflecting more chaotic changes in their brain activity and a more anxious state. This may be related to the changes in brain function caused by COVID-19 infection. In addition, the assessment using the Athens Insomnia Scale showed that the patient’s sleep quality was affected and insomnia symptoms appeared, further supplementing the reason for the increase in SampEn in the delta frequency band. However, we found that the SampEn of patient’s EEG signals in the beta frequency band decreased, this phenomenon may be related to the patient’s decreased attention, thinking activity, and cognitive flexibility.

However, at present, the damage mode of brain network in patients with COVID-19 is not clear. The human brain is the most complex network known to humans, composed of approximately 100 billion (10^11^) neurons interconnected by approximately 100 trillion (10^14^) synapses (Liang et al., 2010). This vast and complex system is the physiological basis for information processing and cognitive expression in the brain, with synapses interacting functionally at multiple time and spatial scales. It is the foundation of our thoughts, feelings, and behaviors. Therefore, studies of the brain can be translated into studies of brain networks. Previous studies have demonstrated that neural imaging data can aid in understanding the state of neurological diseases, as many brain networks of neurological diseases undergo changes. Yu et al. (2020) utilized different frequency bands of electroencephalogram phase synchronization index (PSI) to construct the brain functional network of epilepsy patients. The results indicated that once epilepsy occurred, the patient’s brain network also changed significantly, and this change occurred earlier than the clinical symptoms of epilepsy. And researchers also employed graph theory to quantify the characteristics of the epilepsy brain network and discovered that the local efficiency of the patient’s brain network significantly decreased. Slinger et al. (2022) summarized the study of brain networks of 45 patients with focal epilepsy and found that, compared with the control group, the integration level of the structural network of epilepsy patients was significantly reduced. Except for the clustering coefficient of the β frequency band, there was no significant difference in the functional network between the two groups (Slinger et al., 2022). Recently, the observation of the electroencephalogram signals of COVID-19 patients found that the pattern of changes in the patient’s electroencephalogram signals is highly similar to that of epilepsy patients. Chen et al. (2020) also discovered epileptic-like discharges in the electroencephalogram of two COVID-19 patients. Therefore, it is believed that the application of brain network analysis to COVID-19 patients is also feasible.

After conducting a comparative analysis of EEG signals from COVID-19 patients and healthy control groups, we discovered that COVID-19 can cause varying degrees of decreased signal energy in the prefrontal and temporal regions of the brain across different frequency bands. The energy decrease in the prefrontal region may be linked to cognitive, emotional regulation, and social cognitive impairments, while the energy decrease in the temporal region may indicate abnormalities in language, hearing, memory, and emotion. Additionally, further analysis of the complexity of EEG signals revealed that the sample entropy of patients in the delta frequency band significantly increased, indicating heightened complexity of brain activity and a more chaotic state of mind. Conversely, the sample entropy of the beta frequency band significantly decreased, indicating a reduction in irregularity of brain activity and a possible decrease in attention and cognitive flexibility. These phenomena may be associated with the reorganization of the patient’s brain functional network, the information flow of the patient’s brain network mainly flows from the frontal and occipital regions to the temporal lobe, and the core nodes of the patient’s brain network have been rearranged to the temporal lobe. These results preliminarily demonstrate that COVID-19 patients exhibit certain abnormalities in brain activity during rest.

Due to the impact of the COVID-19, the number of subjects that we can adopt is very limited, the sample size is not large enough, the age of the control group and patients also has some differences. Through literature review, we found that age has no effect on the global efficiency and average clustering coefficient in graph theory analysis (Stanley et al., 2015). Further research could explore the changes in brain activity and topology of patients with COVID-19 along with the course of the disease. I believe that the follow-up research will certainly lay a solid theoretical foundation for the application of artificial intelligence in neurology.

## 5 Conclusion

This EEG study on 15 patients with COVID-19 and 15 healthy people at rest shows that, surprisingly, COVID-19 can significantly reduce the energy in the frontal lobe and temporal lobe of the brain under the four frequency bands of patients, significantly increase the brain activity level of patients in the delta band, significantly reduce the brain activity level of patients in the beta band, and change the brain functional network structure. These results preliminarily demonstrate that COVID-19 patients exhibit certain abnormalities in brain activity during rest, and it is feasible to explore the neurophysiological mechanism of COVID-19’s impact on the nervous system by using EEG signals, which can provide a certain technical basis for the follow-up use of artificial intelligence to predict the prognosis of COVID-19 patients.

## Data availability statement

The original contributions presented in this study are included in this article/supplementary material, further inquiries can be directed to the corresponding author.

## Ethics statement

The studies involving humans were approved by the Ethical Committee of Tianjin First Central Hospital. The studies were conducted in accordance with the local legislation and institutional requirements. Written informed consent for participation in this study was provided by the participants’ legal guardians/next of kin. Written informed consent was obtained from the individual(s) for the publication of any potentially identifiable images or data included in this article.

## Author contributions

YY: Conceptualization, Data curation, Writing – original draft, Formal analysis, Writing – review and editing. YL: Data curation, Formal analysis, Funding acquisition, Investigation, Methodology, Writing – original draft, Writing – review and editing. JY: Writing – review and editing. YC: Software, Writing – original draft. ZG: Resources, Software, Writing – original draft. XL: Formal analysis, Investigation, Methodology, Writing – original draft. SM: Data curation, Writing – original draft. ZW: Writing – review and editing. CZ: Writing – review and editing. DM: Writing – review and editing.
